# An emergency hybrid procedure that combines endoscopic treatment with partial splenic embolization for bleeding esophagogastric varices

**DOI:** 10.1016/j.radcr.2022.02.041

**Published:** 2022-03-19

**Authors:** Fumio Chikamori, Atsuki Maeda, Niranjan Sharma

**Affiliations:** 1 Department of Surgery, Japanese Red Cross Kochi Hospital, 1-4-63-11 Hadaminamimachi, Kochi, 780-8562 Japan; 2 Department of Gastroenterology, Japanese Red Cross Kochi Hospital, 1-4-63-11 Hadaminamimachi, Kochi, 780-8562 Japan; 3 Adv Train Gastroint & Organ Transp Surgery, 12 Scotland Street Dunedin, 9016, New Zealand

**Keywords:** Emergency hybrid procedure, Splanchnic caput Medusae, Esophagogastric varices, Endoscopic injection sclerotherapy, Partial splenic embolization, Polycystic liver disease

## Abstract

Management of splenomegaly is important in the treatment of portal hypertension. We report 2 cases who were treated by an emergency hybrid procedure combining endoscopic treatment and partial splenic embolization (PSE) based on a new concept "splanchnic caput Medusae". Case 1 with refractory esophageal variceal bleeding due to alcoholic liver cirrhosis was treated by endoscopic injection sclerotherapy (EIS) with ligation and PSE at the same time. Case 2 with gastric variceal bleeding due to polycystic liver disease was treated by EIS using n-butyl-2-cyanoacrylate and PSE at the same time. Six days after the hybrid procedure, transjugular retrograde obliteration was added. In both cases, post-treatment 3D-CT reconstruction images revealed that the spleen-portal system reversed to almost normal form. We conclude that an emergency hybrid procedure combining endoscopic treatment and PSE is effective for patients with bleeding esophagogastric varices.

## Introduction

Management of splenomegaly is important in the treatment of portal hypertension. We proposed a new concept: “splanchnic caput Medusae” in which the enlarged spleen is her face and portal collateral pathways are her snake hairs [1]. In the new concept, partial splenic embolization (PSE) is considered as the treatment of Medusae's face. We have reported that PSE not only increases platelet count but also reduces the splenic venous blood flow volume, portal venous pressure, and spleen/liver volume ratio [2,3]. Endoscopic injection sclerotherapy (EIS) and endoscopic variceal ligation (EVL) are considered as the treatment of Medusae's hair. Endoscopic treatment and PSE have been performed on different days so far [1,^4^,5]. Because portal venous pressure is associated with bleeding [6], it is important to reduce portal venous pressure to prevent rebleeding. Here, we report an emergency hybrid procedure that combines endoscopic treatment with PSE for bleeding esophagogastric varices in the digital subtraction angiography (DSA) room at the same time.

Case 1: Bleeding refractory esophageal varices with splenomegaly

A 49-year-old male was referred to the department of surgery at our hospital for refractory esophageal varices and splenomegaly associated with alcoholic cirrhosis. He had a history of hematemesis three times in the past year and was treated endoscopically each time, however, the morphology of esophageal varices was unchanged.

On admission, he did not have jaundice and his consciousness level was lucid. Laboratory studies revealed hemoglobin 14.7 g/dL (normal range, 13.5-17.4); total leukocyte count 3520 /μL (3500 - 8000 /μL); platelet count 7.9 × 10^4^ /μL (12.3 -33.1 × 10^4^ /μL); total bilirubin 1.4 mg/dL (0.3 - 1.3 mg/dL); albumin 4.0 g/dL (3.8 - 5.0 g/dL); aspartate transaminase (AST) 27 U/L (10 - 32 U/L); alanine transaminase (ALT) 28U/L (5 - 27 U/L); prothrombin time (PT) 84.0% (70 -130 %); Mac-2 binding protein glycosylated isomers (M_2_BPGi) 2.06 COI (1+) (<1.00); serum ammonia (NH_3_) 175 μg/dL (12 - 66 μg/dL). Retention rate of indocyanine green at 15 minutes (ICG_15_) was 24 % (<10 %). Child-Pugh score was 5 and the class was A. Hepatitis B surface antigen and hepatitis C virus antibody were negative.

**Fig. 1:**
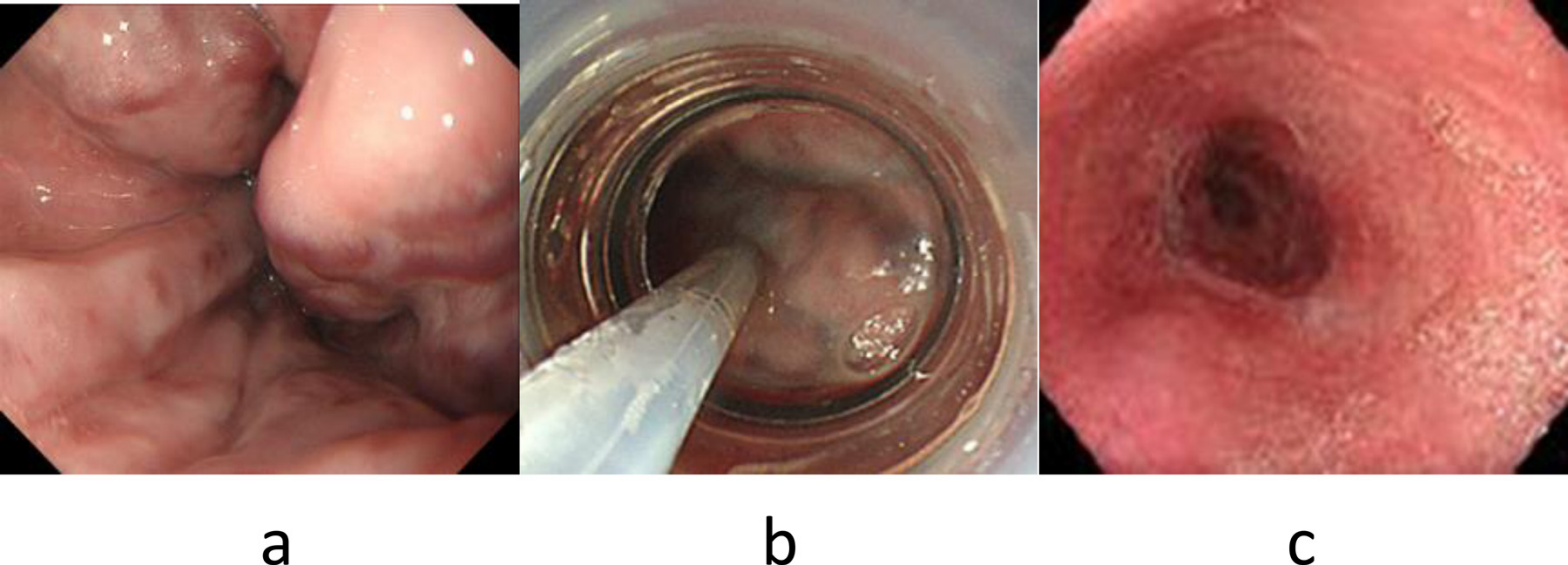
(a). Endoscopy in case 1 shows large and tortuous esophageal varices. (b). Endoscopy shows discolored varices by sclerosant injection during EISL. (c). Endoscopy 6 months after the hybrid procedure shows no esophageal varices.

**Fig. 2:**
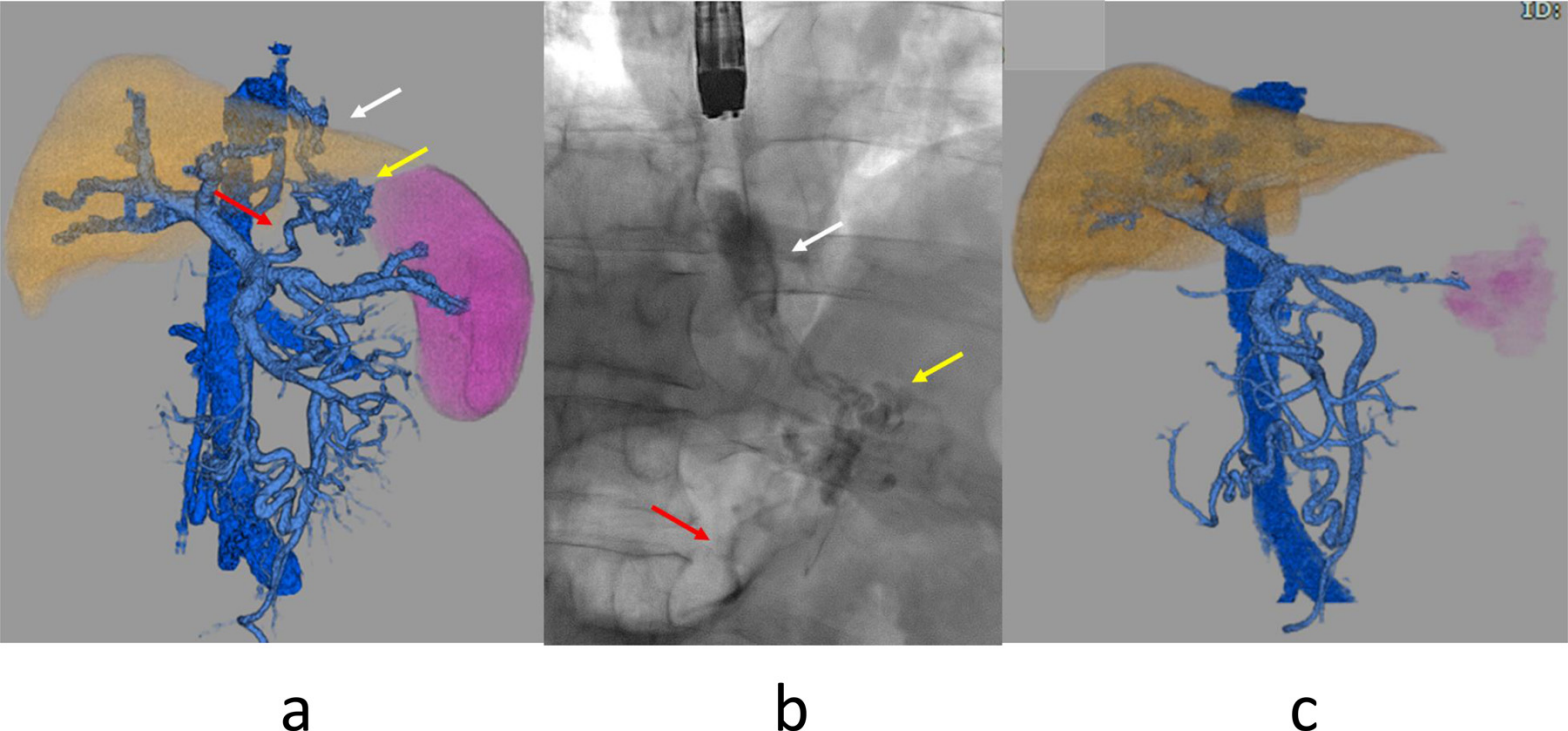
(a). 3D-CT reconstruction image in case 1 shows left gastric vein (red arrow), cardiac venous plexus (yellow arrow), and esophageal varices (white arrow). (b). Endoscopic varicography during EISL shows esophageal varices (white arrow), cardiac venous plexus (yellow arrow), and left gastric vein (red arrow). Blood vessels visualized by EISL match those visualized by 3D-CT. (c). 3D-CT reconstruction image after the hybrid procedure shows a spleen-portal system that has reversed to almost normal form.

Endoscopy confirmed large and tortuous esophageal varices (Fig 1a). Abdominal ultrasonography and contrast-enhanced CT showed splenomegaly. 3D-CT demonstrated that the esophageal varices were supplied by the left gastric vein via the cardiac venous plexus. The spleen volume was 611 ml, the liver volume was 1454 ml; giving a spleen/liver volume ratio [7] of 0.42 (Fig 2a). According to the “splanchnic caput Medusae” concept [1] , the enlarged spleen was regarded as her face and esophageal varices as her snake hairs.

Stepwise PSE [1,2] was attempted to minimize the side effects of PSE. Hepatic venous cannulation was performed. Hepatic venous pressure gradient (HVPG) was 13mmHg (normal range, 1-5mmHg). 1st PSE using gelatin sponge and microcoils was performed. 3D-CT 3 days after 1st PSE revealed that the viable spleen volume decreased to 302 ml and the corrected spleen/liver volume ratio was 0.19.

We recommended the 2nd PSE procedure one month later, but the patient refused for social reasons. Two months later, he was transported to the hospital with hematemesis, and the bleeding from esophageal varices was managed temporarily by EVL. In this case, as the bleeding from esophageal varices was recurrent, an emergency hybrid procedure was performed. Endoscopic injection sclerotherapy with ligation of esophageal varices (EISL) [8] and PSE were performed consecutively under general anesthesia in the DSA room. Total 16 ml of 5% ethanolamine oleate with iopamidol (5%EOI) was injected into the cardiac venous plexus and the root of the left gastric vein in 10 minutes under fluoroscopy (Figs 1b, 2b). The variceal site of injection was ligated immediately after the removal of the needle to stop the variceal blood flow. Subsequently, 2nd PSE was performed and the HVPG was reduced to 8mmHg. 3D-CT one week after the hybrid procedure revealed sufficiently obliterated esophageal varices, cardiac venous plexus, and left gastric vein. The viable spleen volume decreased to 113 ml and the corrected spleen/liver volume ratio was 0.06. One month after 2nd PSE, the platelet count increased to 12.7 × 10^4^ /μL, and he was discharged.

Endoscopy 6 months after the hybrid procedure revealed the distinct collapse of esophageal varices (Fig. 1c). One year after the hybrid procedure, a 3D-CT reconstruction image revealed that the spleen volume was 128 ml and the spleen/liver volume ratio was 0.09. The spleen-portal system reversed to almost normal form (Fig. 2c). The platelet count was 27.7 × 10^4^ / μL. Liver function tests revealed total bilirubin 1.2 mg / dL; albumin 4.2 g / dL; NH_3_ 57 μg/dL; M_2_BPGi 1.07 COI (1+).

Case 2: Bleeding gastric varices in patient with polycystic liver disease

A 68-year-old female was referred to the department of surgery at our hospital for bleeding gastric varices with polycystic liver disease (PLD). Autosomal dominant polycystic kidney disease (ADPKD) was diagnosed at the age of 33, dialysis was introduced at the age of 55, and transcatheter arterial embolization (TAE) [9] was performed at another hospital for PLD at the age of 65.

On admission, she did not have jaundice and her consciousness was lucid. Laboratory studies revealed hemoglobin 6.4 g/dL (normal range, 13.5-17.4); total leukocyte count 5050 /μL (3500 - 8000 /μL); platelet count 14.6 × 10^4^ /μL (12.3 -33.1 × 10^4^ /μL); total bilirubin 0.2 mg/dL (0.3 - 1.3 mg/dL); albumin 2.6 g/dL (3.8 - 5.0 g/dL); AST 9 U/L (10 - 32 U/L); ALT 1U/L (5 - 27 U/L); PT 74.9 % (70 -130 %); M_2_BPGi 0.63 COI (-) (<1.00); NH_3_ 40 μg/dL (12 - 66 μg/dL); blood urea nitrogen (BUN) 128.4 mg/dl (8.0 - 20.0 mg/dL); creatinine (Cre) 7.35 mg/dl (0.36 - 1.06 mg/dL). Child-Pugh score was 7 and the class was B. Hepatitis B surface antigen was negative, but hepatitis C virus antibody was positive.

Abdominal ultrasonography and contrast-enhanced CT showed polycystic liver with calcification and coils used for the previous TAE. 3D-CT demonstrated gastric varices which were supplied by the left gastric vein and drained into the left inferior phrenic vein and gastrorenal shunt. The spleen volume was 372 ml, the liver parenchyma volume was 1873 ml and the total liver volume was 4226ml. Spleen / liver parenchyma volume ratio was 0.20 (Fig. 2a).

**Fig. 3:**
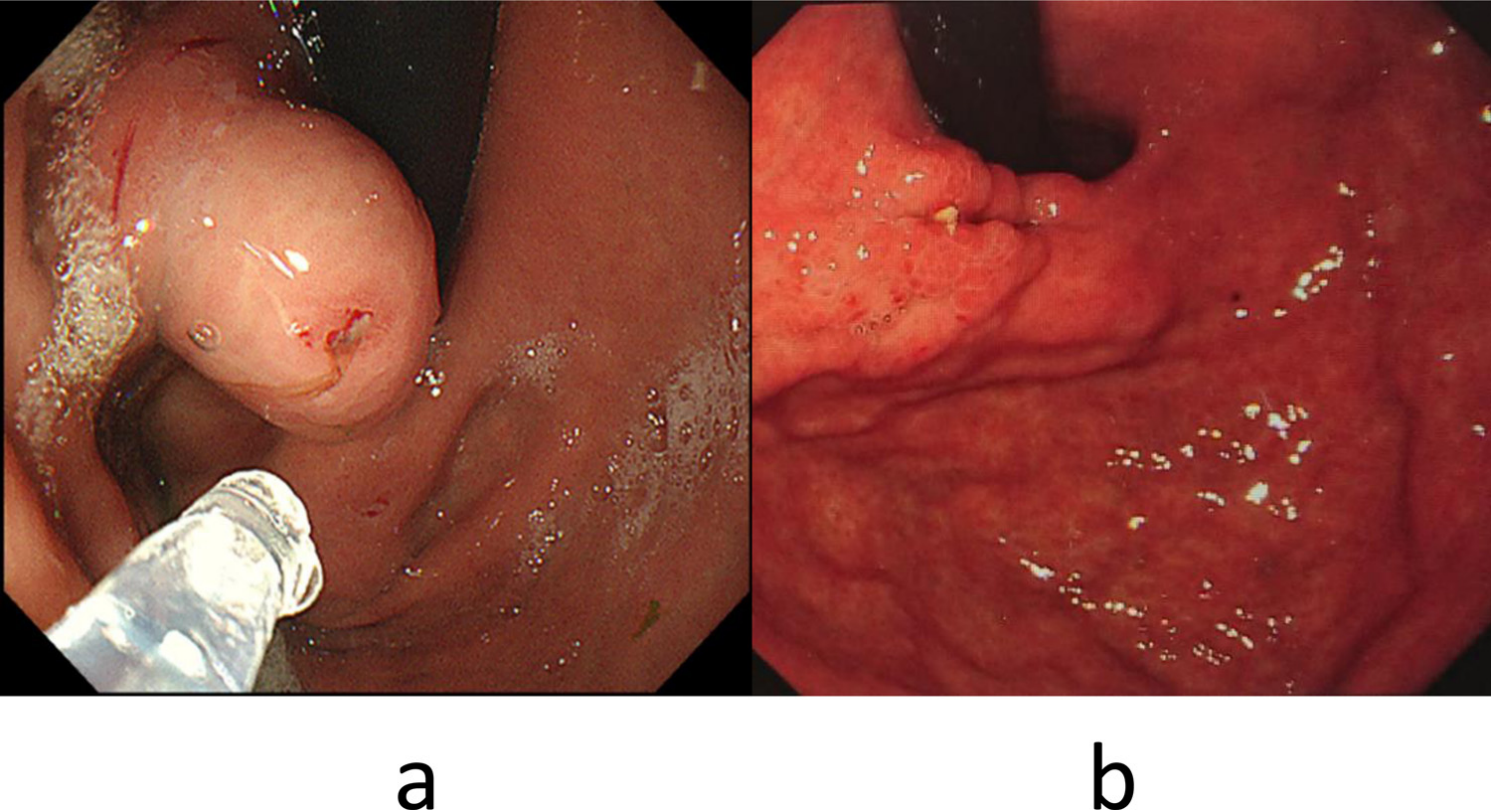
(a). Endoscopy in case 2 shows gastric varix with fibrin-plug. (b). Endoscopy 3 months after TJO following hybrid procedure shows scarred gastric varices.

**Fig. 4:**
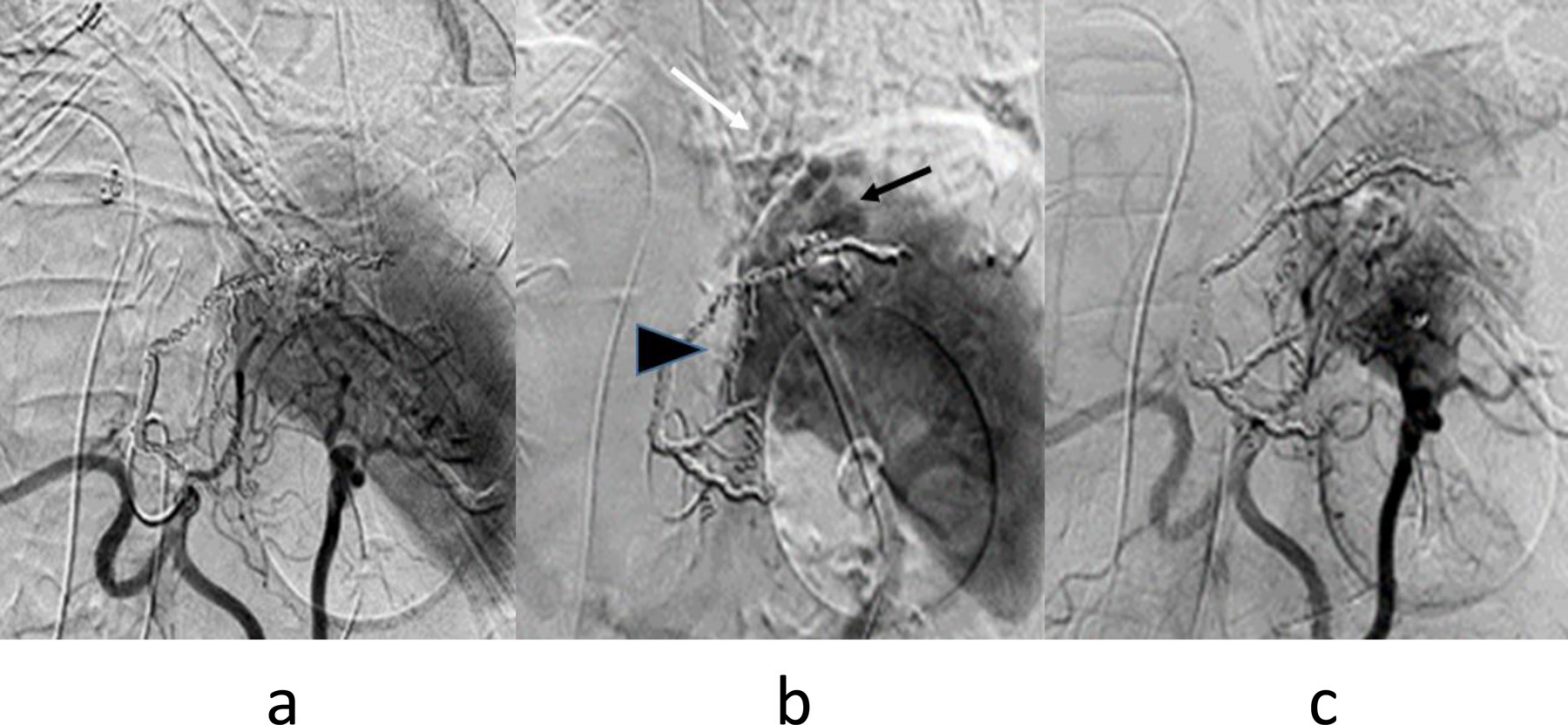
(a). Arterial phase of celiac arteriography in case 2 shows mild splenomegaly. (b). Venous phase of celiac arteriography shows residual gastric varices (black arrow), gastrorenal shunt (arrowhead), and inferior phrenic vein (white arrow). (c). Arterial phase of celiac arteriography after PSE shows 60% embolized spleen.

Endoscopy confirmed gastric varix that was temporarily stopped bleeding by a fibrin plug (Fig. 3a), so an emergency hybrid procedure in the DSA room was performed. EIS using n-butyl-2-cyanoacrylate (NBCA) (Histoacryl) and PSE were performed consecutively under general anesthesia. In EIS, 67% NBCA with ethyl ester of iodinated poppy-seed oil fatty acid (Lipiodol) 1ml in total was injected into gastric varix. Just after removal of the injection needle, one variceal ligation was applied to prevent puncture hole bleeding. Subsequently, PSE with an infarction rate of about 60% was performed (Figs. 4a, b, c).

3D-CT 6 days after the hybrid procedure revealed NBCA-Lipiodol, but the obliteration of gastric varices was insufficient (Fig. 6a). The HVPG was 9 mmHg. In the presence of multiple liver cysts, the reliability of thus obtained value was uncertain, shunt obliteration was considered for the remedy. Transjugular retrograde obliteration (TJO) for gastric varices [10], [11], [12] was attempted by using microcoils, 80 mL 50% glucose, and 5.0 mL absolute ethanol (Fig. 6b).

**Fig. 5:**
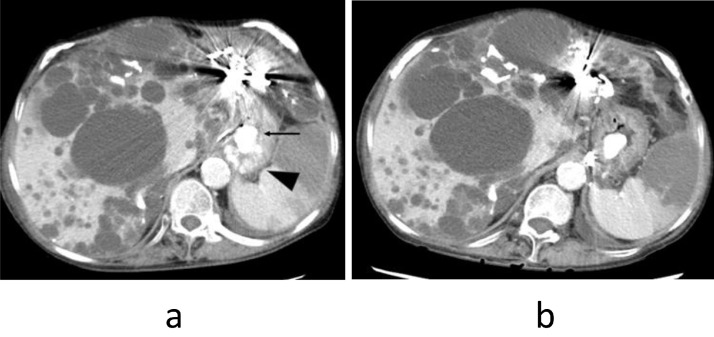
(a). Contrast-enhanced CT after the emergency hybrid procedure in case 2 shows NBCA-Lipiodol (arrow), residual gastric varices (arrowhead), and partially infarcted spleen. (b). Contrast-enhanced CT after TJO shows obliterated residual gastric varices.

**Fig. 6:**
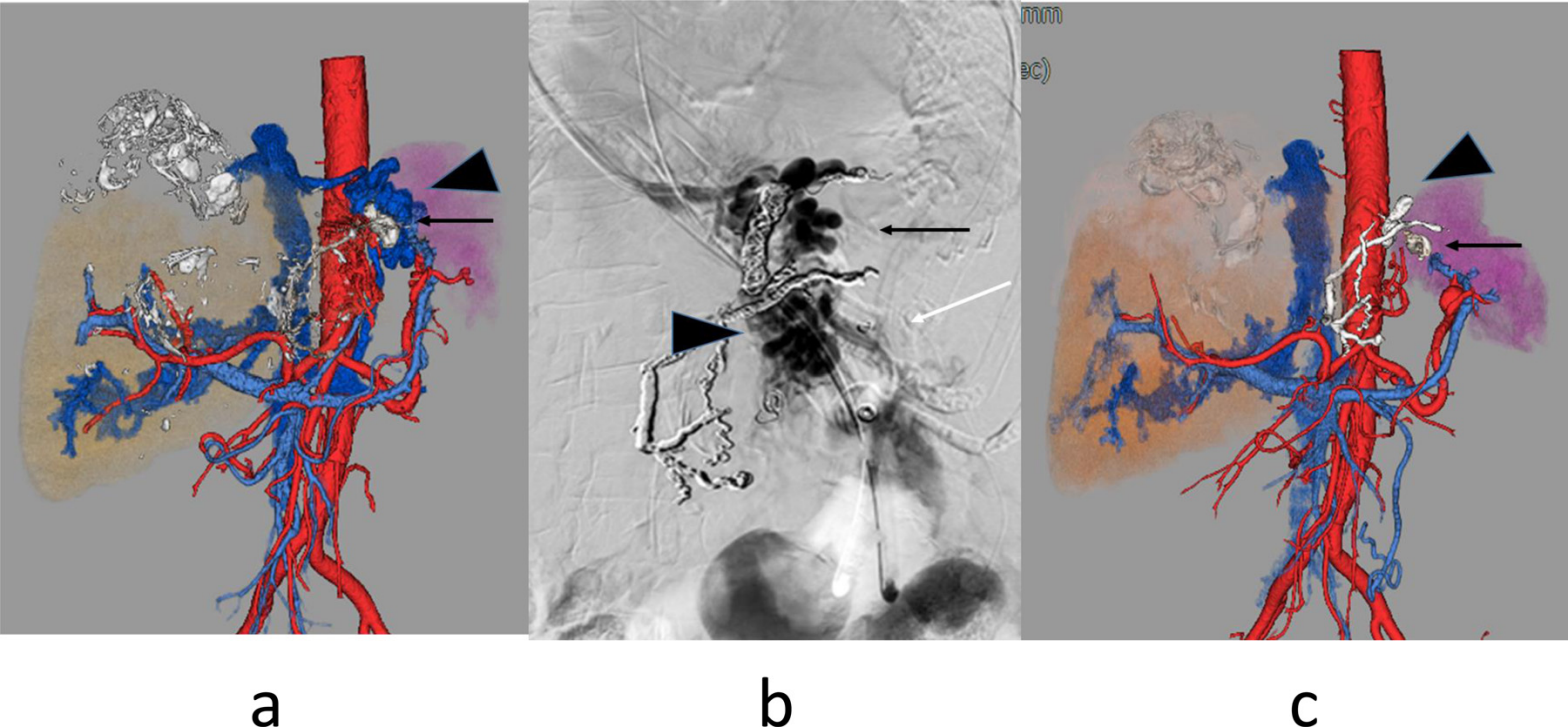
(a). 3D-CT reconstruction image after the emergency hybrid procedure in case 2 shows NBCA-Lipiodol (arrow), residual gastric varices (arrowhead), gstrorenal shunt, and inferior phrenic vein. (b). Retrograde phrenic venography during TJO shows residual gastric varices (arrow), short gastric vein (arrowhead), and peripheral splenic vein (white arrow). (c). 3D-CT reconstruction image after TJO shows NBCA-Lipiodol (arrow), embolized coils in the phrenic vein (arrowhead), no gastric varices, no gastrorenal shunt, and no phrenic vein.

CT scan, nine days after TJO revealed that the gastric varices, inferior phrenic vein, and gastrorenal shunt were successfully obliterated (Fig 5b). The viable spleen volume was 143 ml and the corrected spleen/liver volume ratio was 0.08. The spleen-portal system reversed to almost normal form (Fig 6c).

She was discharged on the 16th day and continued dialysis as an outpatient. Endoscopy 3 months after TJO following hybrid procedure revealed scarred gastric varices (Fig 3b).

## Discussion

As reported, we successfully treated 2 cases of bleeding esophagogastric varices by an emergency hybrid procedure combining endoscopic treatment with PSE at the same time. Case 1 was bleeding refractory esophageal varices with alcoholic cirrhosis and case 2 was bleeding gastric varices with polycystic liver disease.

Hybrid procedures are currently being tried in various fields [13]. In the field of portal hypertension, combined treatment using EIS, EVL, and interventional radiology (IVR) is popular, but each procedure is performed on a different day. This time, in emergency cases of portal hypertension; we performed a hybrid procedure, endoscopic treatment and PSE were performed at the same time.

Currently, endoscopic treatment such as EVL or EIS is the first choice for esophagogastric variceal bleeding. Portal venous pressure is directly correlated with variceal bleeding [6]. To prevent rebleeding, it is important to achieve a reduction in portal venous pressure. PSE not only increases platelet count but also reduces the portal venous pressure, splenic venous blood flow volume and spleen/liver volume ratio [1], [2], [3]. Traditionally, PSE and endoscopic treatment have been performed on different days. The advantage of an emergency hybrid procedure is that hemostasis and portal venous pressure reduction can be expected at the same time. In addition, by performing under general anesthesia, the respiratory movement of the endoscopic puncture target can be controlled, and the injection accuracy can be improved. Even if endoscopic hemostasis fails, it is possible to immediately shift to IVR hemostasis such as percutaneous transhepatic obliteration [14], trans-ileocolic vein obliteration [15], and transjugular intrahepatic portosystemic shunt (TIPS) [16]. If temporary hemostasis is successful as in case 2, time can be secured before permanent hemostasis treatment. Hemodynamics and liver function can be evaluated, and the possibility of shunt obliteration can be examined.

At present, we consider that the indications for hybrid procedures are refractory and/or special types of bleeding esophagogastric varices. The first case was refractory esophageal variceal bleeding with alcoholic cirrhosis who did not follow our treatment schedule. The second was a dialysis patient with rare gastric variceal bleeding with PLD. Causes of portal hypertension in PLD are inferior vena cava syndrome, hepatic venous outflow obstruction, and/or portal vein obstruction because of cystic mass effect [17], [18], [19]. For gastric variceal bleeding, it is necessary to comprehensively determine the treatment plan from portal venous pressure, liver function, consciousness level, ascites, and general condition after temporary hemostasis. Because there was a concern about rebleeding during dialysis, it was necessary to lower the portal venous pressure and reduce the rebleeding rate as much as possible. In this case, HVPG was measured immediately before TJO, and it was 9 mmHg. The value was not so high and might be due to PSE.

There are pros and cons to transjugular intrahepatic portosystemic shunt (TIPS) as a treatment of portal hypertension due to PLD. Regarding TIPS in the presence of cysts, there are reports of contraindications due to the risk of bleeding, but there are also reports of a small number of successful cases [20], [21], [22]. Compared to TIPS, PSE can be expected to reduce portal venous pressure more safely and easily, and the S / L ratio can be improved at the same time.

We did not have a hybrid operating room, so we used the DSA room. Zhao Y, et al [23]. also reported a hybrid procedure combining PSE and balloon occluded endoscopic Histoacryl injection for esophagogastric varices in the DSA room. The current problem for the hybrid procedure is to manage the use of the DSA room. It is also important to collaborate with the endoscopy team, the radiology team, and the anesthesiology team. We conclude that an emergency hybrid procedure combining endoscopic treatment and PSE is feasible and effective for patients with a special type of bleeding esophagogastric varices.

## Patient consent statement

Written informed consent was obtained from the patient for publication of this case report and accompanying images.

## Declaration of Competing Interest

The authors declare no conflicts of interest associated with this manuscript.

## References

[1] Chikamori F, Sharma N, Ito S, Mizobuchi K, Ueta K, Takasugi H, Yukishige S, Matsuoka H, Hokimoto N, Yamai H, Onishi K, Tanida N, Hamaguchi N. (2020). Stepwise partial splenic embolization for portal hypertension based on a new concept: Splanchnic caput Medusae. Radiol Case Rep.

[2] Chikamori F, Kuniyoshi N, Kawashima T, Takase Y. (2007). Short-term portal hemodynamic effects of partial splenic embolization for hypersplenism. Hepatogastroenterology.

[3] Chikamori F, Mizobuchi K, Ueta K, Takasugi H, Yukishige S, Matsuoka H, Hokimoto N, Yamai H, Onishi K, Tanida N, Hamaguchi N, Ito S, Sharma N. (2020). Flood syndrome managed by partial splenic embolization and percutaneous peritoneal drainage. Radiol Case Rep.

[4] Chikamori F, Kuniyoshi N, Kawashima T, Shibuya S, Takase Y. (2004). Combination treatment of partial splenic embolization, endoscopic embolization and transjugular retrograde obliteration for complicated gastroesophageal varices. Hepatogastroenterology.

[5] Chikamori F, Kuniyoshi N, Shibuya S, Takase Y. (2000). Urgent transjugular retrograde obliteration for prophylaxis of rebleeding from gastric varices in patients with a spontaneous portosplenorenal shunt. Dig Surg.

[6] Abraldes JG, Tarantino I, Turnes J, Garcia-Pagan JC, Rodés J, Bosch J (2003). Hemodynamic response to pharmacological treatment of portal hypertension and long-term prognosis of cirrhosis. Hepatology.

[7] Chikamori F, Nishida S, Selvaggi G, Tryphonopoulos P, Moon JI, Levi DM, Kato T, Island ER, Maki A, Tekin A, Tzakis AG. (2010). Effect of liver transplantation on spleen size, collateral veins, and platelet counts. World J Surg.

[8] Nishikawa Y, Y Hosokawa Y, Doi T, Shima S, Miyoshi M, Ohnishi T, Tanimizu M, Hyodo I, Jinno K (1995). Simultaneous combination of endoscopic sclerotherapy and endoscopic ligation for esophageal varices. Gastrointest Endosc.

[9] Hoshino J, Ubara Y, Suwabe T, Sumida K, Hayami N, Mise K, Hiramatsu R, Hasegawa E, Yamanouchi M, Sawa N, Takei R, Takaichi K. (2014). Intravascular embolization therapy in patients with enlarged polycystic liver. Am J Kidney Dis.

[10] Chikamori F, Shibuya S, Takase Y, Ozaki A, Fukao K. (1996). Transjugular retrograde obliteration for gastric varices. Abdom Imaging.

[11] Chikamori F, Kuniyoshi N, Shibuya S, Takase Y. (2001). Eight years of experience with transjugular retrograde obliteration for gastric varices with gastrorenal shunts. Surgery.

[12] Chikamori F, Kuniyoshi N, Kawashima T, Takase Y. (2008). Gastric varices with gastrorenal shunt: combined therapy using transjugular retrograde obliteration and partial splenic embolization. AJR Am J Roentgenol.

[13] Jin H, Liu J. (2020). Application of the Hybrid Operating Room in Surgery: A Systematic Review. J Invest Surg.

[14] Laing IA, Buist TA, Fraser MS (1981). Percutaneous transhepatic occlusion for bleeding oesophageal varices in polycystic disease. Arch Dis Child.

[15] Fujii Y, Sakamori R, Yamada R, Yoshioka T, Kodama T, Shigekawa M, Hikita H, Tanaka S, Ishida H, Mita E, Hongyo H, Higashihara H, Noda T, Eguchi H, Tatsumi T, Takehara T. (2021). The First Transileocolic Obliteration for Refractory Esophageal Varices: A Case Report and Review of the Literature. Intern Med.

[16] Sanyal AJ (2000). The use and misuse of transjugular intrahepatic portasystemic shunts. Curr Gastroenterol Rep.

[17] Bernts LHP, Drenth JPH, Tjwa ETTL (2019). Management of portal hypertension and ascites in polycystic liver disease. Liver Int.

[18] Ratcliffe PJ, Reeders S, Theaker JM (1984). Bleeding oesophageal varices and hepatic dysfunction in adult polycystic kidney disease. Br Med J (Clin Res Ed).

[19] Srinivasan R (1999). Polycystic liver disease: an unusual cause of bleeding varices. Dig Dis Sci.

[20] Spillane RM, Kaufman JA, Powelson J, Geller SC (1997). Waltman AC. Successful transjugular intrahepatic portosystemic shunt creation in a patient with polycystic liver disease. Am J Roentgenol.

[21] Sze DY, Strobel N, Fahrig R, Moore T, Busque S, Frisoli JK (2006). Transjugular intrahepatic portosystemic shunt creation in a polycystic liver facilitated by hybrid cross-sectional/angiographic imaging. J Vasc Interv Radiol.

[22] Shin ES, Darcy MD (2001). Transjugular intrahepatic portosystemic shunt placement in the setting of polycystic liver disease: questioning the contraindication. J Vasc Interv Radiol.

[23] Zhao Y, Wang S, Li C, Guo L, Li C, Zhao L, Tian L, Zheng S, Liu J, Sun G. (2020). Synchronous hybrid procedure combining interventional radiology and endoscopy for esophagogastric varices with large gastro-renal shunt. Medicine.

